# Frustrated Magnetism and Spin Anisotropy in a Buckled Square Net YbTaO_4_

**DOI:** 10.1021/acs.inorgchem.4c04396

**Published:** 2024-12-27

**Authors:** Arun Ramanathan, Martin Mourigal, Henry S. La Pierre

**Affiliations:** † School of Chemistry and Biochemistry, Georgia Institute of Technology, Atlanta, Georgia 30332, United States; ‡ School of Physics, Georgia Institute of Technology, Atlanta, Georgia 30332, United States; § Nuclear and Radiological Engineering and Medical Physics Program, School of Mechanical Engineering, Georgia Institute of Technology, Atlanta, Georgia 30332, United States

## Abstract

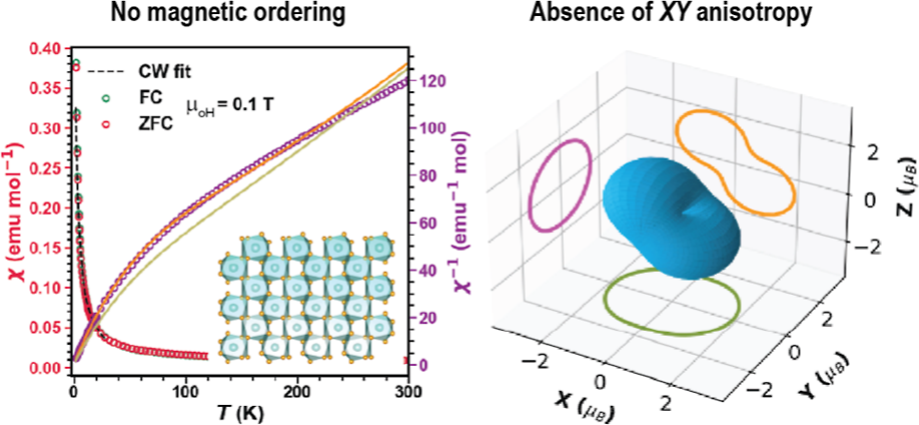

The interplay between quantum effects from magnetic frustration, low-dimensionality, spin–orbit coupling, and crystal electric field in rare-earth materials leads to nontrivial ground states with unusual magnetic excitations. Here, we investigate YbTaO_4_, which hosts a buckled square net of Yb^3+^ ions with *J*_eff_ = 1/2 moments. The observed Curie–Weiss temperature is about −1 K, implying an antiferromagnetic coupling between the Yb^3+^ moments. The heat capacity shows no long-range ordering down to 0.10 K, instead shows a field-dependent broad maximum indicative of short-range correlations. The magnetic entropy recovered and magnetization measurements confirm a spin–orbit driven *J*_eff_ = 1/2 Kramers doublet ground state. Point charge calculations show that the Yb^3+^ ions do not host the quintessential *XY* spin anisotropy observed in typical Yb-based quantum magnets like NaYbO_2_, YbMgGaO_4_, and pyrochlores but rather exhibit an almond-shaped anisotropy with an easy axis. Thus, YbTaO_4_ can serve as a model system to study frustrated magnetism in a square lattice using the *J*_1_–*J*_2_ Heisenberg model. This work also emphasizes the significance of small perturbations to the local crystal electric field that can alter the spin anisotropy and change the collective behavior of the system.

## Abstract 2

We investigate YbTaO_4_ which hosts a buckled square net of *J*_eff_ = 1/2 moments and demonstrate that it lacks long-range magnetic ordering down to 0.1 K and the quintessential *XY* anisotropy.

## Introduction

Quantum spin liquids (QSLs) are exotic magnetic states displaying long-range quantum entanglement and topological order, for instance, in crystalline solids. Electrons’ spins are correlated and entangled in these systems, but conventional magnetic order is absent.^1^ As topological order is accompanied by fractionalized spin excitations, the later provide a route to detect and understand QSLs experimentally.^2^ The search for QSLs has focused on two and three-dimensional frustrated magnets, in which the lattice geometry, further-neighbor exchange interactions and/or bond-dependent interactions from spin–orbit coupling (SOC), introduce frustration and prevent magnetic exchange interactions from being satisfied simultaneously.^3−5^ Suppressed magnetic order, where spins remain strongly fluctuating despite magnetic ordering, can occasionally lead to QSLs, various exotic spin textures, and complex magnetic phase diagram as a function of temperature, magnetic field, and applied pressure.^6,7^ One of the most straightforward routes to stabilize a QSL is starting from the spin-1/2 Heisenberg model on the square lattice, which has been particularly interesting due to its connection with high-temperature superconductivity,^8,9^ introducing frustration through competing nearest and next-nearest neighbor interactions (*J*_1_–*J*_2_ model).^10−12^

When introduced in a magnetically frustrated system, rare-earth (RE) ions with localized 4f electrons host many interesting properties—often stemming from anisotropic exchange interactions— including nontrivial short-range spin correlations, unconventional spin glass, and magnetic Coulomb phases.^13,14^ The delicate interplay among spin–orbit coupling, single-ion anisotropy, exchange interactions, and geometric frustration in rare-earth magnets makes them a rich testbed to realize unique quantum states, including QSLs.^15−18^ Spin–orbit driven quantum magnets have proven to be a fruitful avenue to realize emergent quantum phenomena ranging from spin ice states, anomalous and spontaneous Hall effects to Bose–Einstein condensate phases.^19−21^

A unique attribute of RE magnetism is the hierarchy of energy scales, in which SOC, the crystal field (CF), and exchange interactions have characteristic energies separated by an order of magnitude or more.^22^ The free-ion ground state, found by minimizing the Coulombic and spin–orbit energies in accordance with Hund’s rules, displays a definite total angular momentum *J*. By breaking the rotational symmetry, the CF removes most of the resulting (2*J* + 1)-fold degeneracy.^23^ In the low-energy limit, single-ion physics can often be faithfully described using an effective spin degree of freedom, albeit with an anisotropic *g*-tensor reflecting the spatial anisotropy of the local magnetization distribution.^14^ In turn, exchange interactions between the effective spin degrees of freedom can become anisotropic. The rich quantum magnetic behavior of frustrated RE magnets results from the interplay between single-ion and exchange anisotropies.^13^

In recent years, numerous RE-frustrated quantum magnets have been studied, including triangular and kagome lattice systems.^24−28^ Among them, YbMgGaO_4_ (YMGO) and YbZnGaO_4_ with a triangular lattice of Yb^3+^ ions is the most extensively studied.^27,29−32^ However, quenched chemical disorder due to the mixed occupancies of magnesium and gallium atoms between the magnetic layers introduces *g*-tensor and exchange disorder, likely facilitating the formation of a weakly bound spin-glass state.^27,32^ Fully removing this exchange disorder and accessing the physics inherent to RE quantum magnets remains a challenge, with some recent breakthroughs.^33−37^ Replacing the interlayer cations (Ga and Mg) in YbMgGaO_4_ with a single pentavalent cation (Ta) seems to be an ideal strategy to remove the exchange disorder in the material. However, to retain the triangular lattice motif of Yb^3+^ ions, it is essential for the interlayer Ta^5+^ cations to be supported by ordered vacancies, thereby minimizing the quenched disorder in the material. As a result, replacing Mg and Ga with a single Ta does not stabilize the vacancy-ordered analogue of YbMgGaO_4_. Instead, the system collapses to the thermodynamically stable ortho tantalate YbTaO_4_.^38,39^ One important feature of YbTaO_4_ is that each Yb^3+^ is surrounded by eight oxygen atoms, unlike YbMgGaO_4_ with six neighboring oxygen atoms. The 8-fold coordination is similar to Yb pyrochlore (Yb_2_M_2_O_7_, M = Ti, Ge, Sn) and tripod-kagome (Yb_3_M_2_Sb_2_O_14_, M = Mg, Zn) quantum magnets.^40−42^ While the local oxygen coordination is largely preserved, pyrochlore, tripod-kagome, and YbTaO_4_ exhibit slight perturbations to the ideal coordination environment of the Yb^3+^ ion. These perturbations have been shown to drastically change the single-ion anisotropy of the tripod-kagome system relative to the pyrochlore, yielding different quantum ground states.^43^ This raises an important question of whether the quintessential *XY*-anisotropy observed in pyrochlore is preserved in YbTaO_4_.

To this end, we investigate the magnetic properties of the effective spin-1/2 square-lattice antiferromagnet YbTaO_4_ with a buckled square net of Yb^3+^ ions. RE orthotantalates have been of wide interest because of their luminescent, proton, and oxide-ion conducting and dielectric properties.^44,45^^44−48^ However, the quantum magnetism of YbTaO_4_ remains unexplored. Using a suite of physical property measurements, we show that no spin ordering occurs in this material down to 100 mK despite Kelvin-scale exchange interactions through the short nearest-neighbor bonds [Curie–Weiss (CW) temperature Θ_CW_ = −0.99(1) K]. This indicates a strong degree of magnetic frustration, which we postulate originates from the excellent two-dimensionality of the magnetic Hamiltonian and non-negligible next-nearest neighbor interactions along the square’s diagonal. As a result, YbTaO_4_ is a candidate material for realizing the *J*_1_–*J*_2_ spin-1/2 square lattice antiferromagnet. Furthermore, we use point-charge (PC)-based CF predictions to understand the spin-space anisotropy. We discuss the implications of our results for other known Yb^3+^-based quantum magnets. Furthermore, the PC calculations underscore the significant role of small perturbations to the CF imposed on the localized 4f electrons in driving the single-ion anisotropy and, in turn, the collective spin dynamics of the system.

## Results and Discussion

YbTaO_4_ was synthesized by a conventional solid-state reaction from Yb_2_O_3_ and Ta_2_O_5_ as starting materials. The reaction yields a phase pure white polycrystalline sample, as confirmed by powder X-ray diffraction (Figure S1). The orthotantalates typically crystallize in three common polymorphs: *I*2/*a*, monoclinic (*M*), *I*4_1_/*a*, tetragonal (*T*), and *P*2/*c* monoclinic (*M*′) phases, depending on synthetic conditions.^49−53^ The *T* phase is typically metastable and has not been isolated at room temperature. However, the relative stability of the monoclinic phases depends on the size of the cation with the larger cations (Nd–Er) choosing the *M* phase and the smaller cations (Tm–Lu) choosing the *M*′ phase.^54^ The *M* and *M*′ phases are distinguished only by a change of centering, which means the two phases exhibit different arrangements of the polyhedral building blocks. The *M* phase exhibits a nonlayered arrangement, while the *M*′ phase exhibits distinct layers perpendicular to the *a* axis. The layered structure makes the *M*′ phase a prime candidate for studying quantum magnetism and is the focus of this study. Since the Ln–Ta–O phase space is rich with various metastable phases, isolation of single phase *M*′-YbTaO_4_ depends on the synthetic conditions.

**Figure 1:**
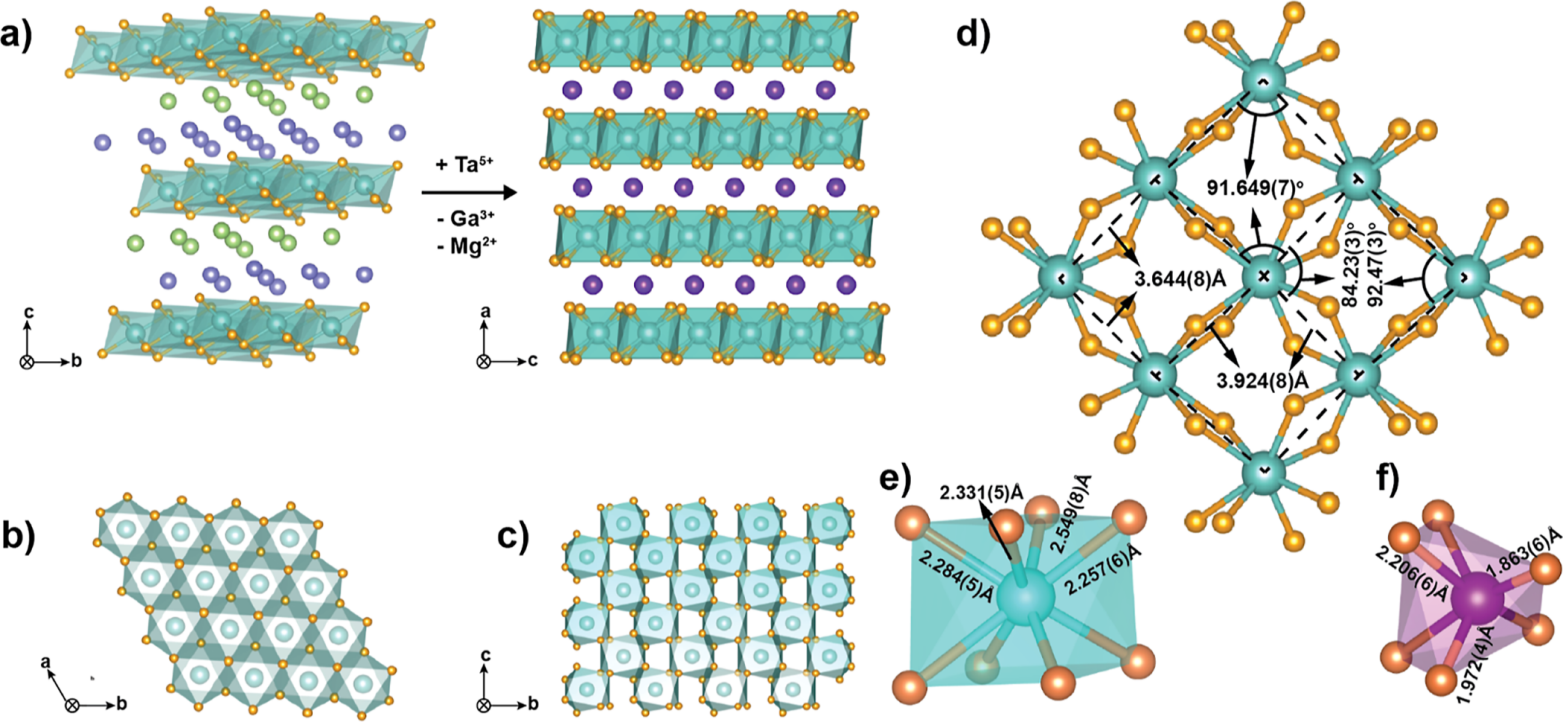
(a) Crystal structure of YbMgGaO_4_ and *M*′-YbTaO_4_. (b) Triangular lattice formed by the Yb^3+^ atoms in YbMgGaO_4_. (c) Buckled square net formed by the Yb^3+^ atoms in *M*′-YbTaO_4_. (d) Buckled square net layer in *M*′-YbTaO_4_, viewed parallel to the crystallographic *a*-axis. All Yb atoms are shown in blue, oxygen in orange, and interlayer atoms in purple. (e) Local coordination for YbO_8_. (f) Local coordination for TaO_6_.

*M*′-YbTaO_4_ crystallizes in the monoclinic *P*2/*c* space group, as confirmed using Rietveld refinement of the PXRD data (Figure S1).^55^ There is only one crystallographic Yb site in *M*′-YbTaO_4_ with the layers stacked along the crystallographic *a* direction. The distance between the two adjacent layers is 8.3501(9) Å, which is much larger than the average distance *d*_Yb–Yb.avg_ = 3.7846(5) Å between the next nearest neighboring Yb ions, making the system quasi-two-dimensional, as shown in Figure 1. The nearest neighbor Yb–Yb distances are relatively short and are only larger than those in NaYbO_2_ (3.35 Å) and YbMgGaO_4_ (3.35 Å) (Table S1). Each layer is composed of Yb polyhedra that share edges with four neighboring Yb polyhedra in the same layer to form a buckled square net with average nearest *∠*Yb–Yb–Yb <90° (Figure 1d). The Ta^5+^ ions are located between the layers and balance the charge. The six oxygen atoms from the Ta ions coordinate to four Yb atoms from one layer and four from the neighboring layer. This leads to a coordination number of eight for every Yb ion with four different Yb–O bond lengths ranging from 2.257(6) to 2.549(6) Å. The polyhedra formed by the Yb ions and the surrounding ligands are shown in Figure 1. This geometry contrasts with the six coordination in NaYbO_2_ and YbMgGaO_4_.^27,34^ The 8-fold coordination is an important structural feature. Even though *M*′-YbTaO_4_ has four distinct Yb–O distances compared to two and three in the pyrochlore and tripod-kagome materials, respectively, the Yb site-symmetry is largely preserved in these materials. Any deviation can be treated as a perturbation in the well-studied pyrochlore case.^13,40^ As a result, the single-ion ground state of YbTaO_4_ is expected to be a  Kramers doublet (KD).

**Figure 2:**
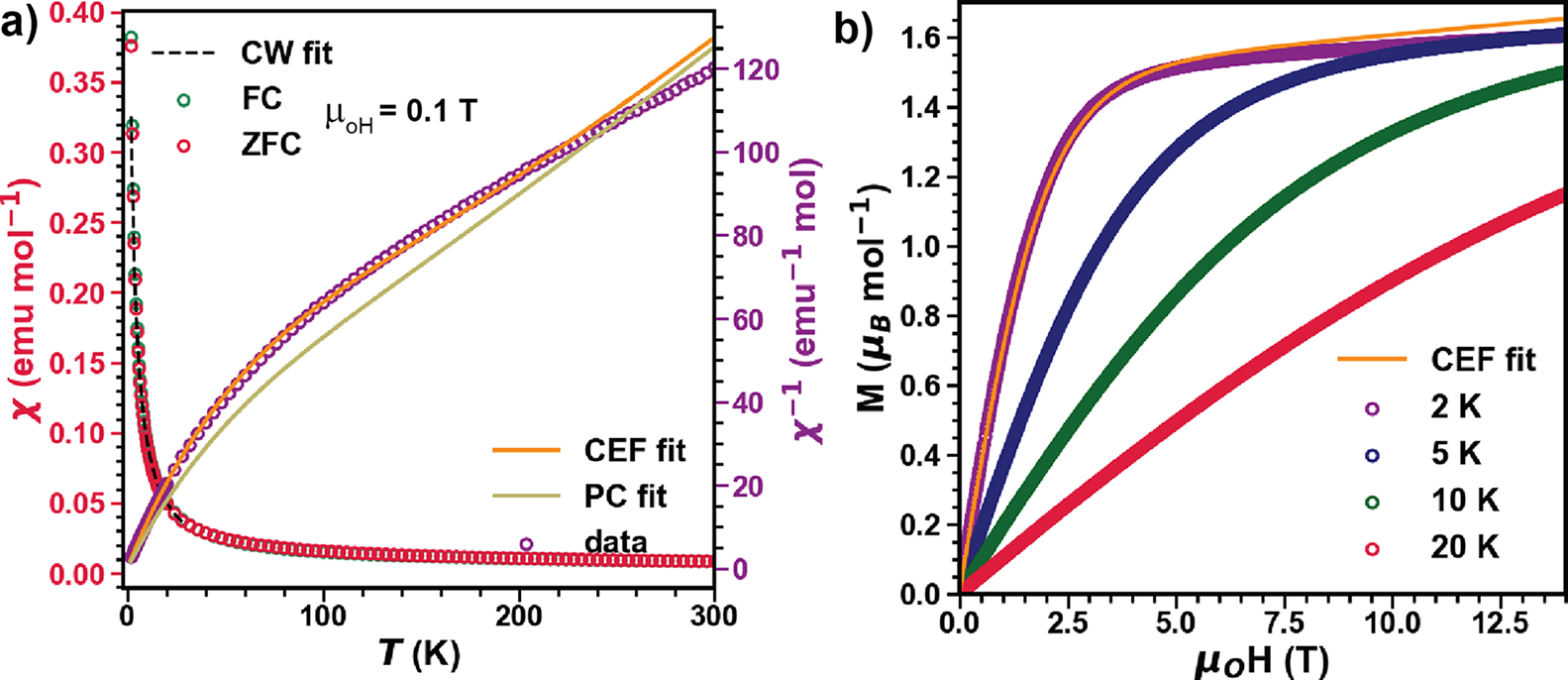
(a) Temperature dependence of magnetic susceptibility (χ(*T*)) measured under μ_0_*H* = 0.1 T in FC and ZFC condition (left axis) and inverse magnetic susceptibility (χ(*T*)^−1^) at the μ_0_*H* = 0.1 T FC condition (right axis) from 1.8 to 300 K in *M*′-YbTaO_4_. The dashed black line is the Curie–Weiss fit from 1.8 to 30 K. The yellow line is the inverse susceptibility obtained from PC calculations (PC fit). The orange line is the modified PC calculation where the susceptibility was used to constrain the CF Hamiltonian (CF fit). (b) Isothermal magnetization *M*(*H*) at different temperatures plotted together with the calculated *M*(*H*) from the CF fit at *T* = 2 K.

The magnetic susceptibility results measured under zero-field cooling (ZFC) and field cooling (FC) conditions show no observable differences and no long-range magnetic ordering down to 1.8 K. The higher temperature data from 300 to 150 K were modeled by linear fits of the form , as shown in Figure 2a, and yield an effective local moment of μ_eff_^HT^ = 4.84(9)μ_B_/Yb^3+^ consistent with the expected value for free Yb^3+^ ions (4.54 μ_B_/Yb^3+^). In this temperature regime, contributions from the CF excitations of Yb^3+^ are expected to contribute to magnetic susceptibility. In the low-temperature regime, the CF excitations can be neglected, as indicated by the slope change of the inverse susceptibility data at around 100 K. A Curie–Weiss fit in that regime yields Θ_CW_^LT^ = −0.99(1) K and μ_eff_^LT^ = 3.102(1)μ_B_ for 1.8 K < *T* < 30 K; the negative value for Θ_CW_^LT^ confirms antiferromagnetic interactions between Yb^3+^ magnetic moments. Θ_CW_^LT^ is significantly less than the CW observed in YbMgGaO_4_ and NaYbO_2_ (Table S1). The extracted μ_eff_^LT^ is close to the value reported for other  Yb quantum magnets and indicates the settling of Yb^3+^ ions into a Kramer’s doublet ground state.^27,34^Figure 2b shows the isothermal magnetization at different temperatures up to 14 T. The magnetization increases rapidly below 4 T, then saturates, and becomes linearly dependent on the field. We obtain a saturated magnetic moment of *M*_s_ = 1.494(2)μ_B_ from which we extract the powder average *g*-factor *g*_avg_ = 2.98 (*M*_s_ = *g*_avg_/2). The saturation field is lower than the *H*_sat_ ≈ 8 and 16 T observed in YbMgGaO_4_ and NaYb*O*_2_, respectively. The observed CW temperature and saturation field semiquantitatively indicate the relatively weak coupling between Yb^3+^ ions compared to the compounds mentioned above. A comparison to other Yb-based quantum magnets is provided in Table S1.

**Figure 3:**
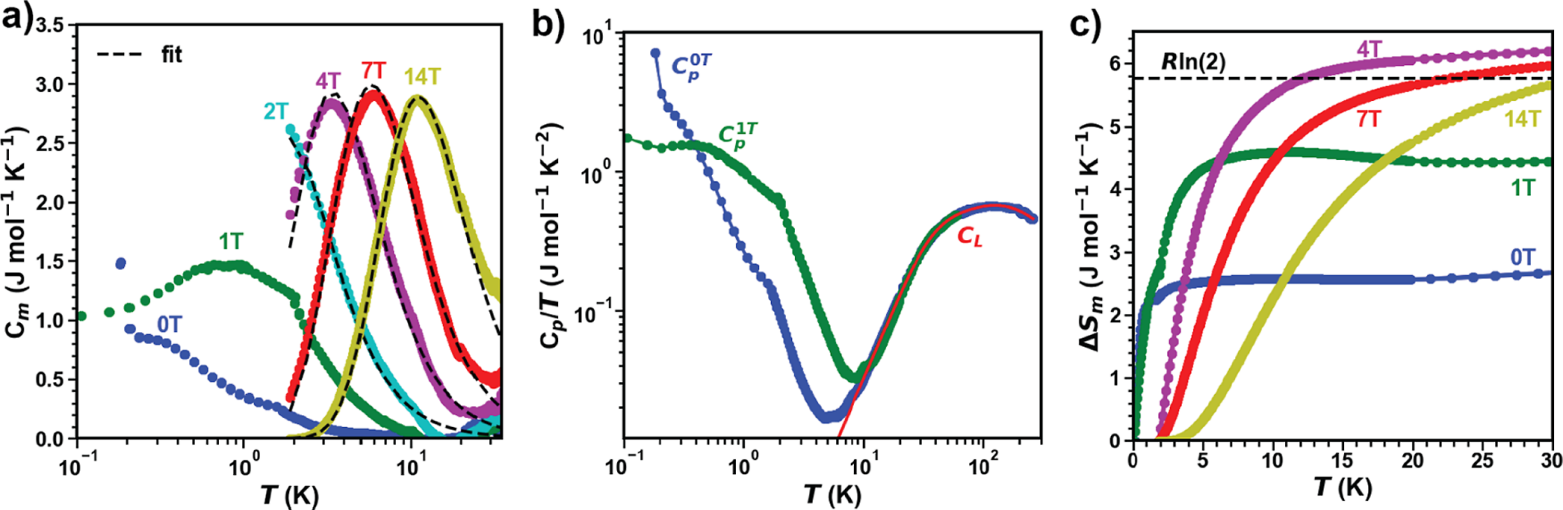
Temperature dependence of the magnetic contribution *C*_m_(*T*) to heat capacity obtained from *C*_p_(*T*) after subtracting the lattice contributions at various magnetic fields from *T* = 0.1 to 30 K, showing a broad peak that moves to higher temperatures with increasing field. The high field data (μ_0_*H* ≥ 2 T) were fit to a two-level Schottky model as shown in the black dotted lines. (b) Temperature dependence of the total specific heat *C*_p_(*T*)/*T* at zero-field and 1 T from 0.1 to 300 K shows the upturn arising from nuclear Schottky contributions. Also plotted is the lattice specific heat obtained from a Debye model (Θ_D1_ ≈ 225 K and Θ_D2_ ≈ 686 K). Note, the small kink observed around 1.8 K comes from two different data sets measured over different temperature ranges (1.8 to 300 K and 0.1 to 1.8 K). (c) Cumulative magnetic entropy [Δ*S*_m_(*T*)] released as a function of temperature at different fields, supporting the assignment of a  ground state.

To further characterize *M*′-YbTaO_4_, heat capacity measurements were performed to probe the low-temperature spin dynamics in *M*′-YbTaO_4_. After estimating the lattice contributions (*C*_L_(*T*)) using a two Debye model (Θ_D1_ ≈ 225 K and Θ_D2_ ≈ 686 K), *C*_m_(*T*) has been extracted (see methods section Specific Heat Analysis for full details). Figure 3a shows *C*_m_(*T*) of *M*′-YbTaO_4_ plotted from 30 to 0.10 K at various fields. An obvious upturn can be observed in *C*_p_/*T*(*T*) plots under zero field and 1 T around 0.15 K, as shown in Figure 3b. This upturn can be attributed to nuclear Schottky contributions from the hyperfine interactions at the ^171^Yb and ^173^Yb nuclei. Therefore, determining the precise nature of zero-field *C*_m_(*T*) is complicated as the nuclear Schottky feature dominates below 0.10 K. However, consistent with susceptibility data, no sharp anomaly indicative of the onset of long-range order is observed in *M*′-YbTaO_4_ and is indicative of a disordered or quantum fluctuating ground-state in *M*′-YbTaO_4_. A broad feature is apparent in *C*_m_(*T*) at 0.50 K in zero field. The broad peak shifts to higher temperatures with increasing magnetic fields, indicating that the low-temperature specific heat is predominantly magnetic in origin, likely indicative of the onset of short-range correlations in the system, and is similar to other Yb-based frustrated magnets.^27,56^ At zero field, the broad feature in *C*_m_(*T*) is reminiscent of short-range correlations, while at finite fields, it could also indicate a Schottky anomaly due to Zeeman splitting of *J*_eff_ = 1/2 ground state KD. Therefore, we fit the peaks to a two-level Schottky model according to equation (1), 
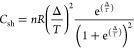
 where *n* is the fraction of free spins, *R* is the gas constant, and Δ is the energy different between the two-level. The corresponding fits are listed in Figure 3a. From a linear fit of the extracted Δ as a function of μ_0_*H* (Δ = *Hgμ*_B_), we extract a *g* factor of ≈2.8 consistent with the value extracted from magnetization (Figure S5). The magnetic entropy change Δ*S*_mag_(*T*) obtained by integrating *C*_m_/*T* over *T* reaches only up to 2.4 J mol^–1^ K^–1^ at zero-field, which is just 42% of the *R*ln 2 expected for  ground state in Yb^3+^, as shown in Figure 3c. This indicates that a residual entropy of 58% needs to be released below 0.10 K, which is a signature of either the low magnetic energy scale of the system or poor thermalization; this similar reduction in entropy has also been observed in a few other Yb-based materials.^24,56−58^ However, at higher fields, Δ*S*_mag_(*T*) reaches close to *R*ln 2 and fully supports the assignment of nominal  magnetic doublet of *M*′-YbTaO_4_ (Figure 3c).This indicates a weak exchange interaction between the Yb^3+^ centers relative to YMGO which recovers 100% *R*ln 2 at zero field at 2 K. Overall, the lack of long-range order and the presence of short-range correlations in the heat capacity data suggest that the *J*_eff_ = 1/2 square-lattice spin system might possess a quantum disordered ground state.

To understand the nature of the ground state observed in *M*′-YbTaO_4_, it is necessary to establish a quantitative understanding of the local moments and microscopic single-ion Hamiltoninan. The Yb^3+^ ions in *M*′-YbTaO_4_ have an electron configuration 4f^13^ with a total angular momentum  ground state and a  excited manifold of states. Under the CF, the 8-fold degenerate  states are split into four KD. The nature of the ground state, the degeneracies, and corresponding eigen energies of the KD’s are determined by the point symmetry and the strength of the CF enforced by the surrounding ligands. Thus, the single-ion physics of Yb^3+^ is primarily determined by the ground state KD which spans the pseudospin variable |±⟩ associated with an effective angular momentum . Our specific heat measurements confirm that the ground state is an isolated  KD. However, to understand the spin-space anisotropy, we use point-charge calculations by which the surrounding ligands are treated as point charges, and their known spatial positions are used to estimate the CF parameters.^59^

**Figure 4:**
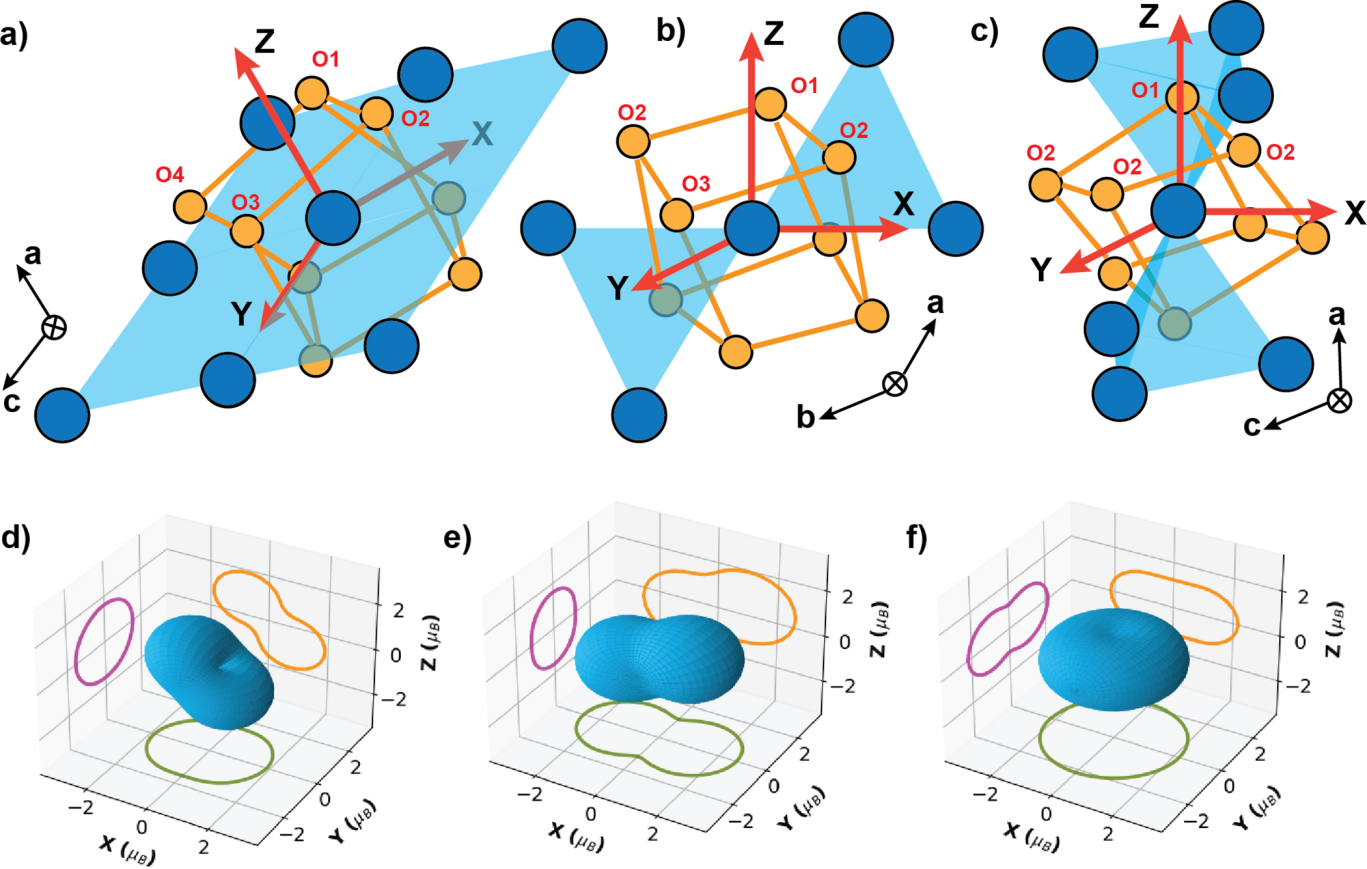
(a) Local coordination environment of Yb^3+^ ions in *M*′-YbTaO_4_ and the buckled square net formed by the neighboring Yb ions. (b) Local coordination environment of Yb^3+^ ions in the tripod-kagome and the kagome net formed by the neighboring Yb ions. (c) Local coordination environment of Yb^3+^ ions in the pyrochlore. All Yb ions are shown in blue, and the oxygen atoms are shown in orange. The red axes at the center show the local axes used to model the CF. (d) Single-ion anisotropy of Yb^3+^ ions in *M*′-YbTaO_4_ shows the almond type *g*-anisotropy with a non-negligible *xz* component. (e) Single-ion anisotropy of Yb^3+^ ions in the tripod kagome shows the almond type *g*-anisotropy. (f) Single-ion anisotropy of Yb^3+^ ions in the pyrochlore shows the continuous *XY* type *g*-anisotropy. The single-ion anisotropies are shown in blue, and the corresponding projections in magenta, orange, and green.

The generalized CF Hamiltonian has the form , where *O*_*n*_^*m*^ are the Stevens’ operators, and *A*_*n*_^*m*^ and *B*_*n*_^*m*^ are the CF parameters.^60^ Constrained by the symmetry of the Yb polyhedra,  was diagonalized using PyCrystalField version 2.3.9 (see methods for details).^61^ However, the PC calculation fails to reproduce the experimental thermo-magnetic data, as shown in Figure 2a. This known discrepancy can be attributed to PC calculations not taking into account the finite extent of the charges on the ions, covalent bonding with the ligands, and the complex effects of “screening” of magnetic electrons by the outer electron shells of the magnetic ion.^59^ Therefore, we adopt a modified PC model, where we use the eight parameters from the PC calculations as a starting point and fit the susceptibility data, thereby constraining the  Hamiltonian. The final fit parameters are reported in Table S7. This yields a model Hamiltonian reproducing the isothermal magnetization at *T* = 2 K. The ground state KD takes the form 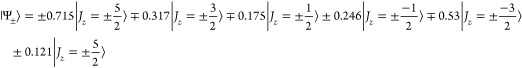 with *g*_*xx*_ = 1.44, *g*_*yy*_ = 2.89, *g*_*zz*_ = 2.18, and a non-negligible off-diagonal term *g*_*xz*_ = 2.89, yielding a powder averaged *g*_avg_^PC^ = 2.61 consistent with *g*_avg_ extracted from magnetization. With *g*_*yy*_ > *g*_*zz*_ > *g*_*xx*_, the single-ion anisotropy of Yb^3+^ in *M*′-YbTaO_4_ has a peanut shape where the *g*-tensor is non-negligible in all three directions, as shown in Figure 4a. This is in sharp contrast to NaYbO_2_, and Yb pyrochlores, which have *XY* anisotropy (Figure 4c), and YbMgGaO_4_, which shows a *XXZ* anisotropy, but peanut-shaped anisotropy has been observed in the tripod-kagome (Figure 4b).^27,43,62,63^

Despite the similar coordination environment of Yb^3+^ ions in the pyrochlore, tripod-kagome, and *M*′-YbTaO_4_ materials, the composition of the respective ground state KD is drastically different. The local coordination of the Yb polyhedra in the pyrochlore, tripod-kagome, and *M*′-YbTaO_4_ and the corresponding single-ion anisotropies are shown in Figure 4. This plot indicates that even though the local coordination is largely preserved in these three materials and that small perturbations of the local symmetry can significantly alter the spin anisotropy and change the collective behavior of the system. The complete list of wave function compositions in all materials is shown in Tables S2–S6. The presence of  component in the ground state KD will have a significant effect in causing quantum tunneling between the Ψ_+_ and Ψ_–_ states. However, the presence of  and  components could also indicate a more classical behavior. Furthermore, the presence of *xz* terms in the *g*-tensor indicates the possible existence of off-diagonal exchange interactions which coupled with SOC of Yb ions could lead to unconventional noncoplanar magnetic spin textures. Thus, *M*′-YbTaO_4_ is the prime candidate for hosting an exotic magnetic quantum ground state.

In summary, *M*′-YbTaO_4_ exhibits magnetic frustration in a square net of Yb^3+^ ions which harbor *J*_eff_ = 1/2 moments. The thermo-magnetic results show no long-range magnetic ordering down to 0.1 K despite the significant antiferromagnetic exchange energy (θ_CW_^LT^ = −1 K) suggesting a quantum disordered ground state. However, the low saturation field and incomplete recovery of magnetic entropy down to 0.10 K under zero-field imply weak quantum entanglement compared to those of the extensively studied YbMgGaO_4_ and NaYbO_2_. Point charge calculations show that the Yb^3+^ ions in *M*′-YbTaO_4_ do not host the quintessential *XY* spin anisotropy observed in typical Yb-based quantum magnets like NaYbO_2_, and pyrochlores but rather exhibits a peanut-shaped anisotropy with an eas*y*-axis. Despite the local coordination of the Yb polyhedra being largely preserved in the pyrochlores, tripod-kagome and *M*′-YbTaO_4_ small perturbations of the CF drastically change the spin-anisotropy. Thus, YbTaO_4_ can serve as a model system to study the frustrated magnetism in a square lattice using the *J*_1_-*J*_2_ Heisenberg model. This work also emphasizes that small perturbations to the local CF in RE^3+^ materials can alter the spin anisotropy and change the collective behavior of the system. While this paper was under review, the independent study of ref (64) appeared with related investigation and similar conclusions as our work.

## Methods

### Materials Synthesis

All reagents were handled in a N_2_ filled glovebox (Vigor) with O_2_ < 0.1 ppm and H_2_O < 0.1 ppm. Yb_2_O_3_ (99.99%, Alfa Aesar) and Ta_2_O_5_ (99.99%, Alfa Aesar) were used as the starting materials. The metal oxide powders were dried by heating to 500 °C for 12 h with a heating rate of 10 °C/min in a box furnace (using alumina crucibles) under an ambient atmosphere. The reagents were then cooled with the furnace off to 120 °C and then cooled to room temperature in the antechamber of the glovebox under a vacuum. These dried reagents were stored in amber bottles in the glovebox. All crucibles were purchased from MTI.

Polycrystalline powder samples of *M*′-YbTaO_4_ were synthesized using traditional solid-state methods by intimately mixing Yb_2_O_3_ and Ta_2_O_5_ in a 1:1 molar ratio (Yb:Ta), using an agate mortar inside the glovebox. The powder mixtures were pressed in to 15 mm diameter pellets outside the glovebox. The firing was performed at 1300 °C for 24 h with a cooling/heating rate of 3 °C/min. The samples were ground, and the above procedure was repeated three times. The phase purity was found to be 97 wt % with a minority Yb_2_O_3_ phase from the starting material.

### Powder X-Ray Diffraction

Laboratory powder X-ray diffraction (PXRD) was collected on a PANalytical X’Pert PRO Alpha-1 diffractometer with Cu *Kα* source in reflection geometry equipped with a fixed divergence slit of 1/8^*″*^, a convergence slit of 1/4^*″*^, and a working radius of 240 mm. The samples were homogenized by finely grinding them inside the glovebox using an agate mortar for about 15 min. To avoid the exposure of the sample to atmospheric air, a PANalytical domed sample holder was equipped with a stainless steel base and a polycarbonate dome with a 70% X-ray transmission. A 2Θ range of 5–85*°* was used with a scan speed of 5 s and a step size of 0.1.

### Physical Property Measurements

The dc magnetic susceptibility measurements and isothermal magnetization measurements were performed using a Quantum Design Physical Properties Measurement System. The sample was sealed in a plastic capsule on a brass holder. The heat capacity was measured at different magnetic fields using a Quantum Design Physical Properties Measurement System. The sample was pressed into pellets. A small pellet yielding 3.3 mg was used for the measurement. For 0.1 K ≤ *T* ≤ 1.8 K, a Quantum Design dilution fridge insert was employed. The sample was ground together with 50% by mass of silver and pressed into pellets. A small pellet yielding 2.25 mg was used for the measurement.

### Point Charge Calculations

The generalized CEF Hamiltonian has the form , where *O*_*n*_^*m*^ are the Stevens’ operators and *A*_*n*_^*m*^ and *B*_*n*_^*m*^ are the CEF parameters.^60^ This formalism is convenient when the ligand environment has a high symmetry, leaving only a handful of CEF parameters to be fit. Unfortunately, the low symmetry of the Yb polyhedra in *M*′-YbTaO_4_ requires eight parameters. Fitting eight parameters without knowing the eigen energies of the CEF states is not feasible. Therefore, we begin with a constrained fit based on an electrostatic point charge model of the ligand environment where we treat the electrostatic field at the Yb site generated by the coordinating oxygen atoms as point charges.^59^ This approximation is valid only for insulating materials like *M*′-YbTaO_4_. We first chose our local axes, as shown in Figure 4 of the main text, to ensure that all imaginary CEF terms are zero. All CEF parameters for the three compounds were calculated using the ligand positions from the corresponding CIF’s. This is referred to as the PC fit in the main text. However, the model does not reproduce the experimental thermo-magnetic data, as shown in Figure 2 of the main text. As a second step, the effective charges of the symmetry-independent ligand sites were refined by fitting the experimental magnetic susceptibility data. There are eight ligands surrounding each Yb^3+^ ion but only four symmetry independent ligand sites. So, the effective charges of each symmetry-independent atoms and the relative weights of each symmetry-related group of ligands were fit starting with effective charges of (−2*e*, – 2*e*, – 2*e*) for O^2–^ ions. The fitting procedure was minimized using χ^2^. All fitting and diagonalization were carried out using PyCrystalField version 2.3.9.^61^ The single-ion anisotropies were visualized by calculating the magnetization along each axes.

### Specific Heat Analysis

The lattice contributions to the total heat capacity were calculated according to the following Debye equation with two different contributions yielding four different variables as shown in equation 2 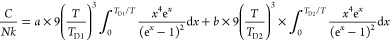 where *T*_D1_ and *T*_D2_ are the Debye temperatures, *k* is the Boltzmann constant, and *a* and *b* are the coefficients.^65^ The final fit results are shown in Figure S4. The fit yields *a* = 1.764(4), *T*_D1_ = 226.801(9) K, *b* = 4.190(6), and *T*_D2_ = 686.877(3) K.

## Supporting Information Available

PXRD Rietveld refinements, Debye fits to specific heat, heat capacity data, and CF and PC calculations showing the CF levels and the wave functions (PDF)
