# Supplementary material for: Frustrated Magnetism and Spin Anisotropy in a Buckled Square Net YbTaO4
Source: Inorg Chem. 2024 Dec 27;64(1):158–65. doi: 10.1021/acs.inorgchem.4c04396 (PMC11734108; doi:10.1021/acs.inorgchem.4c04396)
Supplement: Supplementary file 1 — ic4c04396_si_001.pdf [file ic4c04396_si_001.pdf]

Frustrated Magnetism and Spin Anisotropy in a Buckled Square Net $YbTaO_4$

Arun Ramanathan,[†] Martin Mourigal,[‡] and Henry S. La Pierre[*,¶,§]

†Department of Chemistry, Columbia University, New York, NY 10027, USA

‡School of Physics, Atlanta, GA 30332, USA

¶School of Chemistry and Biochemistry, Atlanta, GA 30332, USA

§Nuclear and Radiological Engineering and Medical Physics Program, School of Mechanical Engineering, Atlanta, GA 30332, USA

E-mail: hsl@gatech.edu

Supplementary Materials

1 Powder X-ray diffraction (PXRD). S2

2 Point charge calculations. S2

3 Specific heat analysis. S9

References S11

1 Powder X-ray diffraction (PXRD).

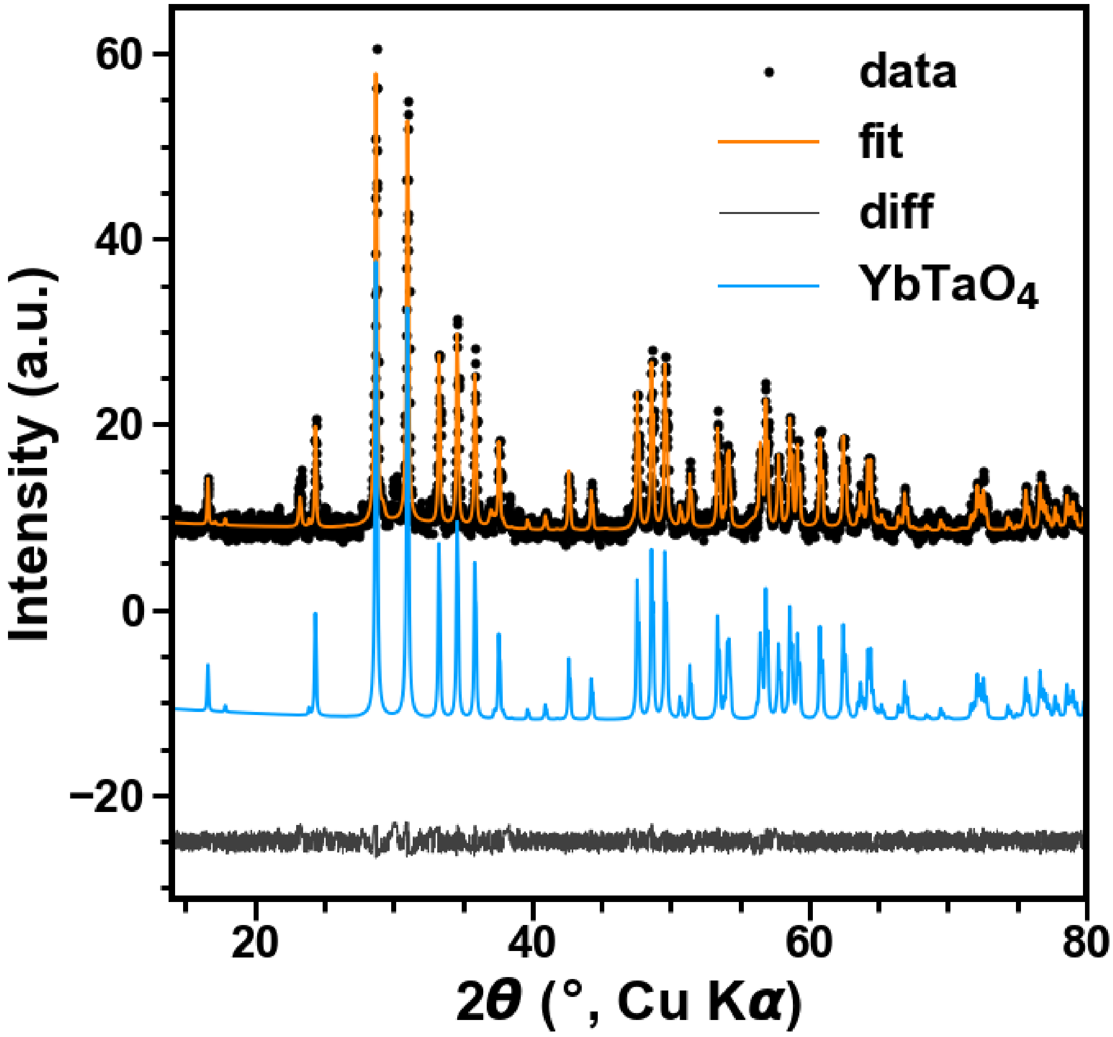

Figure S1: PXRD of M'-$YbTaO_4$. pattern for Data is shown in black dots, Rietveld refinements in orange, the corresponding phases in blue, and the difference curves in grey. All data was collected at $T = 300$ K.[1] Quantitative Rietveld refinements to the laboratory XRD data were carried out using Bruker TOPAS 5 suite.[2]

2 Point charge calculations.

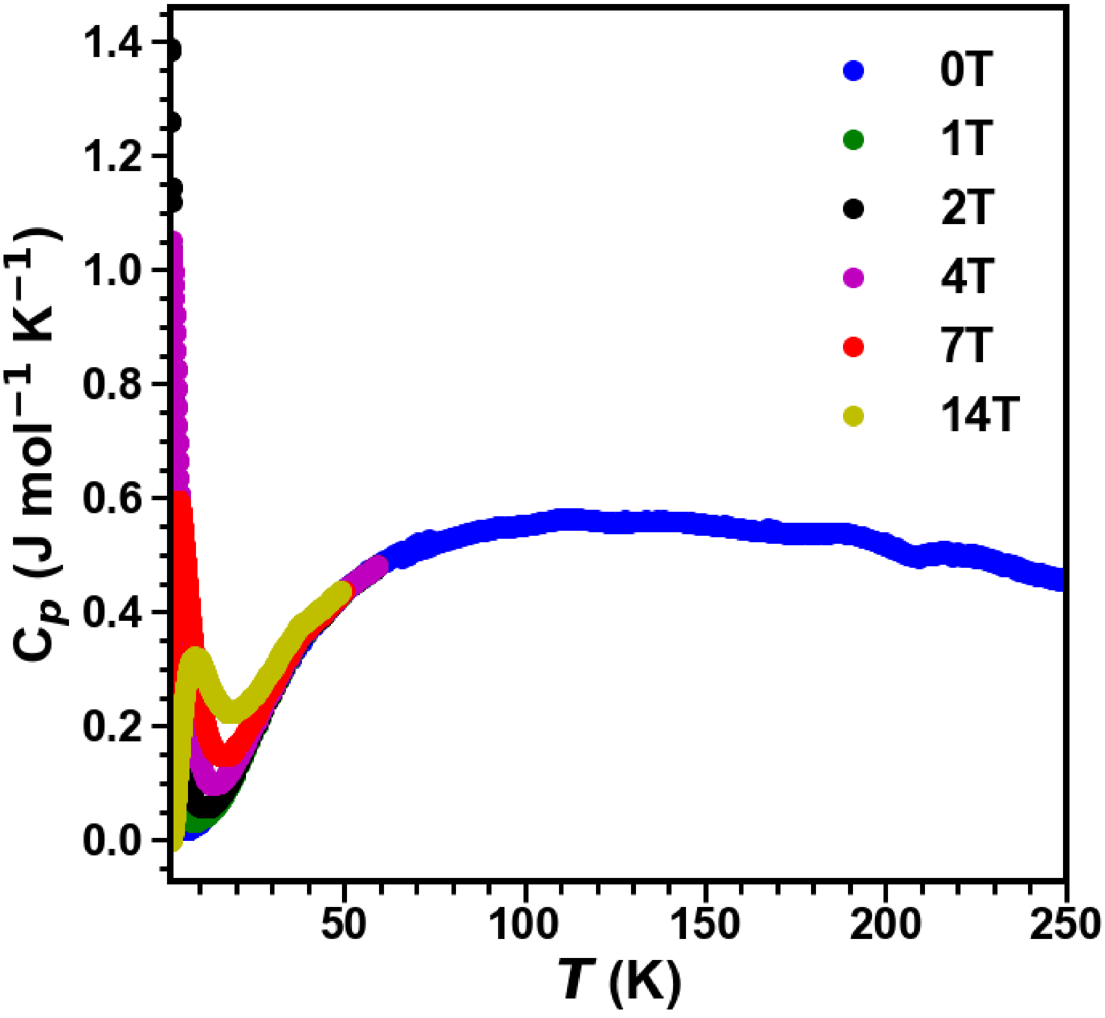

Figure S2: Temperature dependence of the total specific heat $C_p(T)$ at different fields showing no lamda like anomaly.

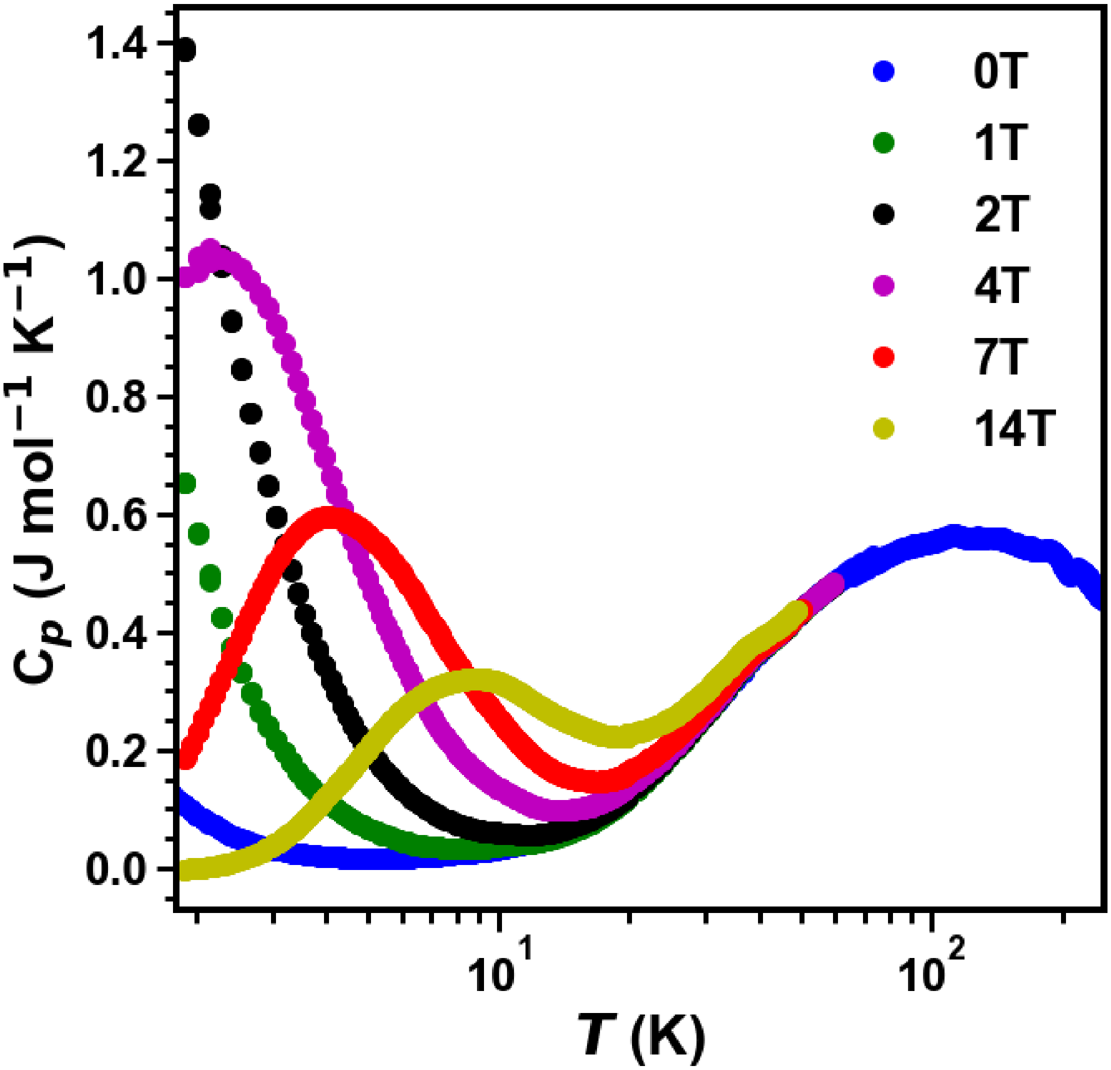

Figure S3: Temperature dependence of the total specific heat $C_p(T)$ at different fields plotted in log scale.

Table S1: Comparison of Structural and Magnetic Features of Ytterbium based quantum magnets.

Material	Avg. Yb-Yb distance (Å)	θ_{CW} (K)	g-anisotropy	Ref
M'-$YbTaO_4$	3.78	-0.986	Almond with easy axis	This work
$Yb_2Ti_2O_7$	3.6	0.8	XY	[3]
$YbMgGaO_4$	3.40	-4.11	XXZ	[4]
$NaYbO_2$	3.34	-5.64	XY	[5]
$Yb_3Mg_2Sb_3O_{14}$	3.63	-0.39	Almond with easy axis	[6]
Bi_2YbO_4Cl	3.82	-1	Easy axis	[7]
$YbBO_3$	3.75	-0.28	-	[8]
$RbBaYb(BO_3)_2$	5.41	-0.08	-	[9]
$LiYbSe_2$	3.97	-3.14	-	[10]
$KYb_2F_5SO_4$	3.88	-0.46	-	[11]
$KYbSe_2$	-0.0359	-14	XY	[12]

Table S2: Eigenvectors and eigenvalues for M'-$YbTaO_4$ from the PC fit.

E (meV)	$\vert -\frac{7}{2}\rangle$	$\vert -\frac{5}{2}\rangle$	$\vert -\frac{3}{2}\rangle$	$\vert -\frac{1}{2}\rangle$	$\vert \frac{1}{2}\rangle$	$\vert \frac{3}{2}\rangle$	$\vert \frac{5}{2}\rangle$	$\vert \frac{7}{2}\rangle$
0.000	0.0	-0.0367	-0.2363	-0.1542	-0.0949	-0.1037	0.9049	0.2837
0.000	-0.2837	0.9049	0.1037	-0.0949	0.1542	-0.2363	0.0367	0.0
28.825	0.8693	0.2216	-0.1054	0.2749	0.0382	-0.3264	0.0017	-0.0216
28.825	0.0216	0.0017	0.3264	0.0382	-0.2749	-0.1054	-0.2216	0.8693
38.975	0.2521	0.322	-0.3205	-0.1985	-0.0816	0.8048	-0.0756	0.1747
38.975	0.1747	0.0756	0.8048	0.0816	-0.1985	0.3205	0.322	-0.2521
65.479	-0.2607	0.1266	-0.2246	0.832	-0.3897	0.1219	0.0726	-0.0359
65.479	-0.0359	-0.0726	0.1219	0.3897	0.832	0.2246	0.1266	0.2607

Table S3: Eigenvectors and eigenvalues for M'-$YbTaO_4$ from the CEF fit1.

E (meV)	$\|-\frac{7}{2}\rangle$	$\|-\frac{5}{2}\rangle$	$\|-\frac{3}{2}\rangle$	$\|-\frac{1}{2}\rangle$	$\|\frac{1}{2}\rangle$	$\|\frac{3}{2}\rangle$	$\|\frac{5}{2}\rangle$	$\|\frac{7}{2}\rangle$
0.000	-0.1084	0.0	-0.5104	0.0	0.079	0.0	0.8494	0.0
0.000	0.0	0.8494	0.0	0.079	0.0	-0.5104	0.0	-0.1084
29.438	0.0	0.3124	0.0	-0.3811	0.0	0.2864	0.0	0.8217
29.438	0.8217	0.0	0.2864	0.0	-0.3811	0.0	0.3124	0.0
43.722	0.3163	0.0	-0.8072	0.0	-0.2687	0.0	-0.4197	0.0
43.722	0.0	-0.4197	0.0	-0.2687	0.0	-0.8072	0.0	0.3163
53.520	-0.4616	0.0	0.0765	0.0	-0.8811	0.0	0.069	0.0
53.520	0.0	-0.069	0.0	0.8811	0.0	-0.0765	0.0	0.4616

Table S4: Eigenvectors and eigenvalues for M'-$YbTaO_4$ from the CEF final fit.

E (meV)	$\|-\frac{7}{2}\rangle$	$\|-\frac{5}{2}\rangle$	$\|-\frac{3}{2}\rangle$	$\|-\frac{1}{2}\rangle$	$\|\frac{1}{2}\rangle$	$\|\frac{3}{2}\rangle$	$\|\frac{5}{2}\rangle$	$\|\frac{7}{2}\rangle$
0.000	-0.0282	0.1207	-0.5299	0.2464	-0.1755	-0.3174	0.7152	0.0
0.000	0.0	0.7152	0.3174	-0.1755	-0.2464	-0.5299	-0.1207	-0.0282
40.820	-0.0019	0.1115	0.0556	0.3438	-0.2065	0.1379	-0.0856	0.8929
40.820	0.8929	0.0856	0.1379	0.2065	0.3438	-0.0556	0.1115	0.0019
41.216	0.0059	0.4707	-0.425	0.4552	-0.0451	0.3593	-0.4025	-0.3121
41.216	-0.3121	0.4025	0.3593	0.0451	0.4552	0.425	0.4707	-0.0059
41.394	-0.3231	-0.1607	0.087	0.5262	0.5114	-0.5282	-0.2114	-0.009
41.394	0.009	-0.2114	0.5282	0.5114	-0.5262	0.087	0.1607	-0.3231

Table S5: Eigenvectors and eigenvalues for $Yb_2Ti_2O_7$ from PC calculations.

E (meV)	$\|-\frac{7}{2}\rangle$	$\|-\frac{5}{2}\rangle$	$\|-\frac{3}{2}\rangle$	$\|-\frac{1}{2}\rangle$	$\|\frac{1}{2}\rangle$	$\|\frac{3}{2}\rangle$	$\|\frac{5}{2}\rangle$	$\|\frac{7}{2}\rangle$
0.000	-0.3742	0.0	0.0	-0.9214	0.0	0.0	0.1053	0.0
0.000	0.0	0.1053	0.0	0.0	0.9214	0.0	0.0	-0.3742
38.615	0.0	0.0008	0.0	0.0	-0.3764	0.0	0.0	-0.9265
38.615	-0.9265	0.0	0.0	0.3764	0.0	0.0	0.0008	0.0
47.109	0.0	0.0	0.2183	0.0	0.0	-0.9759	0.0	0.0
47.109	0.0	0.0	0.9759	0.0	0.0	0.2183	0.0	0.0
75.140	0.0404	0.0	0.0	0.0972	0.0	0.0	0.9944	0.0
75.140	0.0	0.9944	0.0	0.0	-0.0972	0.0	0.0	0.0404

Table S6: Eigenvectors and eigenvalues for $Yb_3Mg_2Sb_3O_{14}$ from PC calculations.

E (meV)	$\|-\frac{7}{2}\rangle$	$\|-\frac{5}{2}\rangle$	$\|-\frac{3}{2}\rangle$	$\|-\frac{1}{2}\rangle$	$\|\frac{1}{2}\rangle$	$\|\frac{3}{2}\rangle$	$\|\frac{5}{2}\rangle$	$\|\frac{7}{2}\rangle$
0.000	0.0	-0.1239	-0.0358	-0.0871	-0.6197	0.0224	-0.0296	0.7683
0.000	0.7683	0.0296	0.0224	0.6197	-0.0871	0.0358	-0.1239	0.0
27.335	-0.0011	0.113	0.1062	0.0937	0.7293	0.2235	0.0604	0.6179
27.335	0.6179	-0.0604	0.2235	-0.7293	0.0937	-0.1062	0.113	0.0011
44.688	-0.1595	-0.1915	0.9264	0.1129	-0.0729	0.033	-0.2419	-0.0439
44.688	-0.0439	0.2419	0.033	0.0729	0.1129	-0.9264	-0.1915	0.1595
60.036	0.0227	-0.4769	-0.2752	-0.1333	0.1785	0.043	-0.803	0.007
60.036	-0.007	-0.803	-0.043	0.1785	0.1333	-0.2752	0.4769	0.0227

Table S7: CEF parameters for different models of M'-$YbTaO_4$. Also shown is the parameters for the pyrochlore and tripod-kagome.

CF $B_m^n(meV)$	PC fit	CEF fit1	Final CEF fit	Pyrochlore PC	Tripod Kagome PC
B_2^{-2}	0.0	0.0	0.0	0.0	0.0
B_2^{-1}	0.0	0.0	0.0	0.0	0.0
B_2^0	-0.754	0.107	0.165	0.535	-0.349
B_2^1	-0.109	0.058	0.253	0.0	-0.345
B_2^2	0.641	0.538	0.141	0.0	-0.515
B_4^{-4}	0.0	0.018	0.0	0.0	0.0
B_4^{-3}	0.0	-0.030	0.0	0.0	0.0
B_4^{-2}	0.0	0.010	0.0	0.0	0.0
B_4^{-1}	0.0	-0.018	0.0	0.0	0.0
B_4^0	0.029	0.0	0.015	-0.044	-0.033
B_4^1	-0.082	-0.015	0.012	0.0	0.023
B_4^2	0.004	0.001	0.004	0.0	-0.006
B_4^3	0.288	0.006	-0.113	0.334	0.345
B_4^4	0.073	0.007	0.111	0.0	0.026
B_6^{-6}	0.0	0.0	0.0	0.0	0.0
B_6^{-5}	0.0	0.0	0.0	0.0	0.0
B_6^{-4}	0.0	0.0	0.0	0.0	0.0
B_6^{-3}	0.0	0.0	0.0	0.0	0.0
B_6^{-2}	0.0	0.0	0.0	0.0	0.0
B_6^{-1}	0.0	0.0	0.0	0.0	0.0
B_6^0	0.0	0.0	0.0	0.0	0.0
B_6^1	0.0	0.0	0.0	0.0	0.0
B_6^2	0.0	0.0	0.0	0.0	0.0
B_6^3	0.0	0.0	0.0	0.0	0.0
B_6^4	0.0	0.0	0.0	0.0	0.0
B_6^5	0.0	0.0	0.0	0.0	0.0
B_6^6	0.0	0.0	0.0	0.0	0.0

3 Specific heat analysis.

The lattice contributions to the total heat capacity was calculated according to the following Debye equation with two different contributions yielding four different variables, $\frac{C}{Nk} = a * 9(\frac{T}{T_{D1}})^3 \int_0^{T_{D1}/T} \frac{x^4 e^x}{(e^x-1)^2}\, dx + b * 9(\frac{T}{T_{D2}})^3 \int_0^{T_{D2}/T} \frac{x^4 e^x}{(e^x-1)^2}\, dx$, where T_{D1} and T_{D2} are the Debye temperatures, k is the Boltzman constant, and a and b are the coefficients.[13] The final fit results are shown in Figure S4. The fit yields $a = 1.764(4)$, $T_{D1} = 226.801(9)K$, $b = 4.190(6)$, and $T_{D2} = 686.877(3)K$.

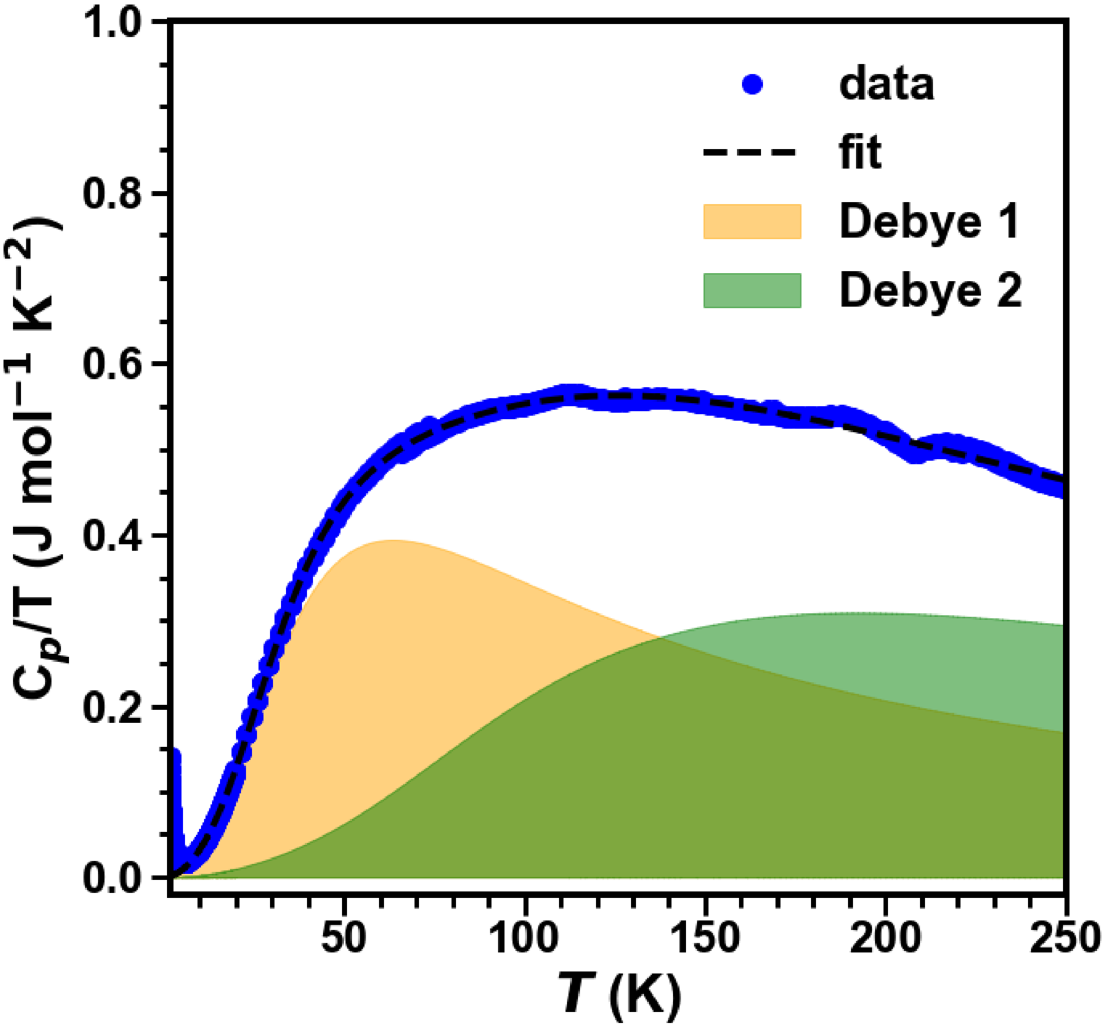

Figure S4: Temperature dependence of the total specific heat $C_p(T)$ at zero field plotted with the two Debye lattice fit.

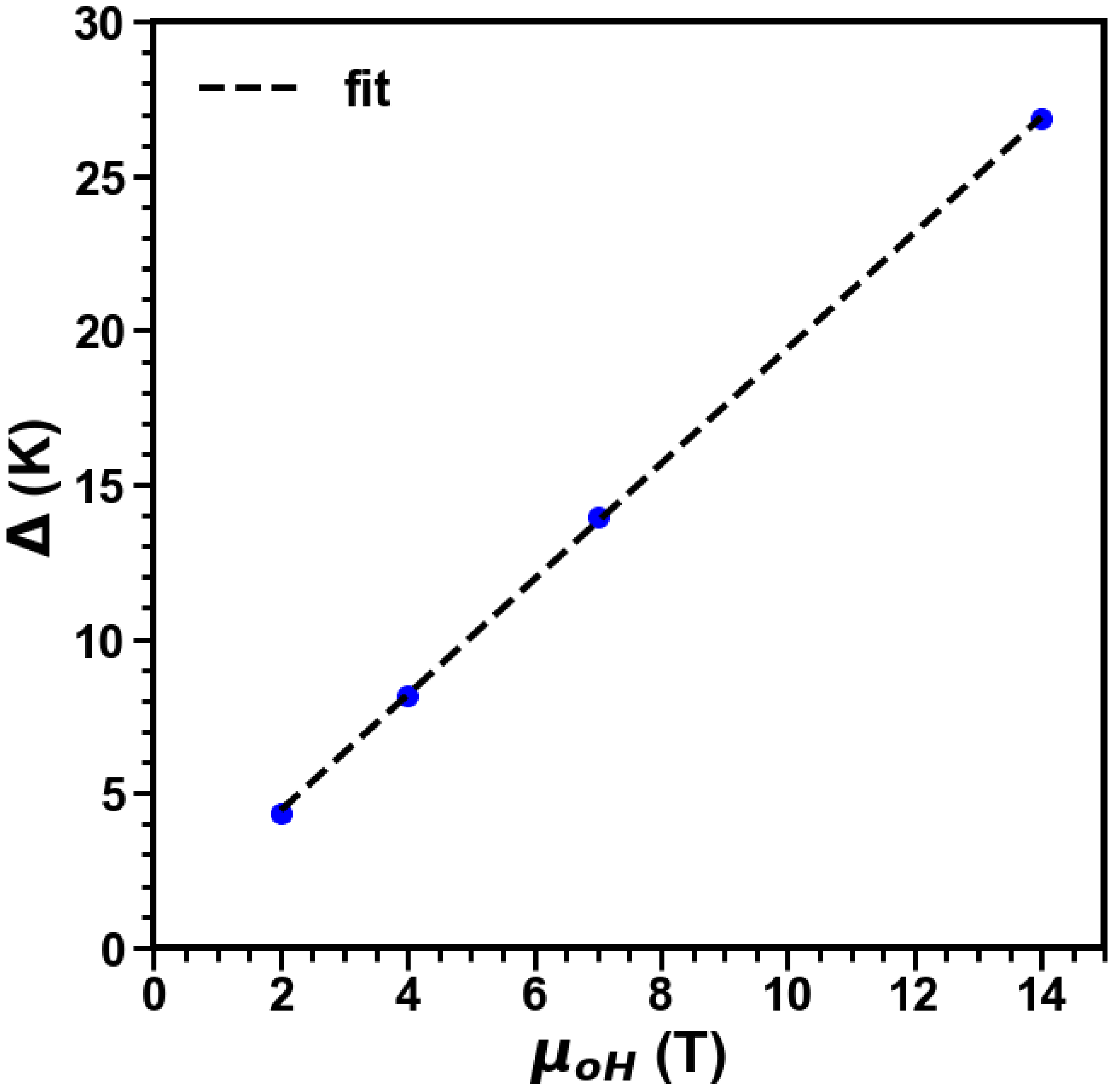

Figure S5: Field dependence of Δ extracted from the two-level Schottky fitting as described in the main text. The dashed line corresponds to the linear fit used to extract the g factor.

References

(1) Rietveld, H. M. A profile refinement method for nuclear and magnetic structures. *Applied Crystallography* **1969**, *2*, 65–71

.

(2) Coelho, A. A. TOPAS and TOPAS-Academic: an optimization program integrating computer algebra and crystallographic objects written in C++. *Journal of Applied Crystallography* **2018**, *51*, 210–218

.

(3) Ross, K.; Proffen, T.; Dabkowska, H.; Quilliam, J.; Yaraskavitch, L.; Kycia, J.; Gaulin, B. Lightly stuffed pyrochlore structure of single-crystalline $Yb_2Ti_2O_7$ grown by the optical floating zone technique. *Physical Review B—Condensed Matter and Materials Physics* **2012**, *86*, 174424

.

(4) Paddison, J. A.; Daum, M.; Dun, Z.; Ehlers, G.; Liu, Y.; Stone, M. B.; Zhou, H.; Mourigal, M. Continuous excitations of the triangular-lattice quantum spin liquid $YbMgGaO_4$. *Nature Physics* **2017**, *13*, 117–122

.

(5) Bordelon, M. M.; Kenney, E.; Liu, C.; Hogan, T.; Posthuma, L.; Kavand, M.; Lyu, Y.; Sherwin, M. S.; Butch, N. P.; Brown, C. M.; Wilson, S. D. Field-tunable quantum disordered ground state in the triangular-lattice antiferromagnet $NaYbO_2$. *Nature Physics* **2019**, *15*, 1058–1064

.

(6) Dun, Z.; Trinh, J.; Li, K.; Lee, M.; Chen, K.; Baumbach, R.; Hu, Y.; Wang, Y.; Choi, E.; Shastry, B., et al. Magnetic ground states of the rare-earth tripod kagome lattice $Mg_2RE_3Sb_3O_{14}$ (RE= Gd, Dy, Er). *Physical Review Letters* **2016**, *116*, 157201

.

(7) Singh, V.; Nam, K.; Barik, M.; Boya, K.; Kermarrec, E.; Khuntia, P.; Kim, K. H.; Bhowal, S.; Koteswararao, B. Bi_2YbO_4Cl: A two-dimensional square-lattice compound with $J_{eff} = 1/2$ magnetic moments. *Physical Review B* **2024**, *109*, 075128

.

(8) Somesh, K.; Islam, S.; Mohanty, S.; Simutis, G.; Guguchia, Z.; Wang, C.; Sichelschmidt, J.; Baenitz, M.; Nath, R. Absence of magnetic order and emergence of unconventional fluctuations in the $J_{eff} = 1/2$ triangular-lattice antiferromagnet $YbBO_3$. *Physical Review B* **2023**, *107*, 064421

.

(9) Guo, S.; Kong, T.; Xie, W.; Nguyen, L.; Stolze, K.; Cevallos, F. A.; Cava, R. Triangular Rare-Earth Lattice Materials $RbBaR(BO_3)_2$ (R= Y, Gd-Yb) and Comparison to the KBa R (BO3) 2 Analogs. *Inorganic Chemistry* **2019**, *58*, 3308–3315

.

(10) Dissanayaka Mudiyanselage, R. S.; Wang, H.; Vilella, O.; Mourigal, M.; Kotliar, G.; Xie, W. $LiYbSe_2$: Frustrated Magnetism in the Pyrochlore Lattice. *Journal of the American Chemical Society* **2022**, *144*, 11933–11937

.

(11) Jiang, N.; La Pierre, H. S. Frustrated magnetism in a 2-D ytterbium fluoride. *Inorganic Chemistry* **2019**, *58*, 12152–12156

.

(12) Xing, J.; Sanjeewa, L. D.; May, A. F.; Sefat, A. S. Synthesis and anisotropic magnetism in quantum spin liquid candidates $AYbSe_2$ (A= K and Rb). *APL Materials* **2021**, *9*

.

(13) Debye, P. Zur theorie der spezifischen wärmen. *Annalen der Physik* **1912**, *344*, 789–839

.
